# Complete Genome Sequence of the Cryptophycin-Producing Cyanobacterium *Nostoc* sp. Strain ATCC 53789

**DOI:** 10.1128/MRA.00040-20

**Published:** 2020-04-02

**Authors:** Anna Tippelt, Tobias Busche, Christian Rückert, Markus Nett

**Affiliations:** a Department of Biochemical and Chemical Engineering, TU Dortmund University, Dortmund, Germany; b IIT Biotech GmbH Bielefeld, Bielefeld University, Bielefeld, Germany; c Technology Platform Genomics, Center for Biotechnology (CeBiTec), Bielefeld University, Bielefeld, Germany; University of Southern California

## Abstract

*Nostoc* sp. strain ATCC 53789 is a producer of cryptophycins, which are promising anticancer agents. Here, we report the completely sequenced 8.7-Mb genome of *Nostoc* sp. strain ATCC 53789. The sequence provides insights into the metabolic network of this cyanobacterial strain and illuminates its potential for the biosynthesis of secondary metabolites.

## ANNOUNCEMENT

The cyanobacterium *Nostoc* sp. strain ATCC 53789 produces two classes of bioactive secondary metabolites, i.e., the cryptophycins (1, 2) and the nostocyclopeptides (3). The cryptophycins were found to be potent anticancer agents (4, 5), which led to their clinical testing (6). Although the biosynthesis genes for cryptophycins and nostocyclopeptides were previously identified (7, 8), their integration into the metabolic network of the producing strain remained unclear.

To complement the existing information, the genome of *Nostoc* sp. strain ATCC 53789 was sequenced and assembled. Unless otherwise specified, default parameters were used for all software. The required DNA was isolated by phenol-chloroform extraction from a culture grown in BG-13 medium (1) under diurnal illumination for 1 month directly after receipt of the strain. The genome was reconstructed from short- and long-read DNA data sets obtained by Illumina and Nanopore sequencing. Library preparation involved a TruSeq DNA PCR-free high-throughput library prep kit (Illumina) and the SQK-LSK109 ligation sequencing kit (Oxford Nanopore Technologies [ONT]). Illumina sequencing was performed using a MiSeq reagent kit v3 (600 cycle) in a 2 × 300-nucleotide (nt) run. For Nanopore sequencing, a GridION platform with an R9.4.1 flow cell was used. Base calling and demultiplexing were performed using Guppy v3.1.5. Illumina data were assembled with Newbler v2.8 (9) (options: -large, -siom 16, -m, –consed). Nanopore data were processed with Canu v1.8 (10) (parameters: genomeSize = 6m, rawErrorRate = 0.3, correctedErrorRate = 0.1). Canu contigs were polished with Racon v1.3.3 (11) (parameters: -c 6, -m 8, -x -6, -g -8, -w 500), followed by medaka v0.11.0 (12) (parameters: -b 100, -m r941_min_high_g303) and Pilon v1.22 (13). minimap2 v2.17 (parameters: -ax sr, –secondary = no), BWA-MEM v2 (14) (parameters: -O1, -E1), and Bowtie 2 v2.3.2 (15) (parameters: -X 750, –no-unal) were used for mapping. Unicycler v0.4.6 (16) was used for hybrid assembly of the Illumina data and the contigs from the polished Canu assembly.

The assemblies were combined manually in Consed v27.0 (17). First, the chromosome was reoriented based on the *dnaA* gene. Overlapping ends from the polished Canu assembly were trimmed by assembly in Consed. Ambiguities in these regions as well as all other repeat regions were corrected based on the contigs produced by the Newbler assembly. Finally, all differences between the contigs of the three assemblies as well as low-quality regions marked in Consed were resolved by manual curation using IGV v2.4.14 (18) for visualization of the ONT data. This approach led to the identification of 13 replicons with a total size of 8,653,729 bp, including 1 circular chromosome, 10 circular plasmids, and 2 linear plasmids (Table 1). The plasmid topology was assessed by Canu and Unicycler and subsequently verified by inspection of the assemblies in Consed using the Illumina reads and the included to/fm/pr information provided by Newbler. The reads were mapped back onto the assembled contigs/replicons and checked in IGV for potential misassemblies. Genome annotation with Prokka v1.11 (19) resulted in the assignment of 7,408 genes, 7,300 protein-coding sequences, 88 tRNAs, 12 rRNAs, and 8 noncoding RNAs (ncRNAs).

**TABLE 1:** Genomic features of *Nostoc* sp. strain ATCC 53789

Replicon	Length (bp)	Topology	G+C content (%)	Coverage (×) for:		No. of biosynthetic loci^*a*^
				Nanopore data	Illumina data	
chr	7,340,101	Circular	41.4	298	39	18
pNsp_a	337,072	Circular	41.6	397	54	1
pNsp_b	325,114	Circular	40.9	330	43	1
pNsp_c	219,529	Circular	41.0	314	41	1
pNsp_d	65,222	Linear	41.8	314	62	1
pNsp_e	57,504	Circular	42.5	418	48	0
pNsp_f	56,077	Linear	41.7	528	98	0
pNsp_g	54,032	Circular	42.3	503	70	0
pNsp_h	49,561	Circular	39.6	361	61	0
pNsp_i	40,105	Circular	41.2	663	103	0
pNsp_j	38,437	Circular	41.4	450	94	0
pNsp_k	36,221	Circular	40.7	341	74	0
pNsp_l	34,754	Circular	42.0	349	64	0

a According to antiSMASH.

An antiSMASH v5.0.0 (20) analysis revealed a distinctive secondary metabolome comprising 22 loci. Interestingly, the cryptophycin locus (7) resides on plasmid pNsp_c, flanked by transposase genes. Moreover, the analysis indicated that the strain is capable of anabaenopeptin biosynthesis (21).

### Data availability.

The annotated nucleotide sequences of the chromosome and the 12 plasmids of *Nostoc* sp. strain ATCC 53789 have been deposited at GenBank under the accession numbers CP046703, CP046704, CP046705, CP046706, CP046707, CP046708, CP046709, CP046710, CP046711, CP046712, CP046713, CP046714, and CP046715. The raw data are available in the SRA under the accession numbers SRR10969384 and SRR10969385.
